# Cardiac Imaging for Risk Assessment of Malignant Ventricular Arrhythmias in Patients With Mitral Valve Prolapse

**DOI:** 10.3389/fcvm.2021.574446

**Published:** 2021-02-15

**Authors:** Bhupendar Tayal, Francesca N. Delling, Maan Malahfji, Dipan J. Shah

**Affiliations:** ^1^Division of Cardiovascular Imaging, Houston Methodist DeBakey Heart and Vascular Institute, Houston, TX, United States; ^2^Department of Cardiolgy, Aalborg University Hospital, Aalborg, Denmark; ^3^Department of Cardiolgy, University of California, San Francisco, San Francisco, CA, United States

**Keywords:** mitral valve prolapse, cardiac imaging, ventricular tachycardia, echocardiagraphy, cardiac magnet resonance imaging

## Abstract

Recent studies have described the occurrence of complex ventricular arrhythmias and sudden cardiac death among patients with mitral valve prolapse (MVP). The reported incidence rate of sudden cardiac death or ventricular tachycardia is about 1–1.5% among patients with MVP. Various imaging markers have been associated with this increased risk, including mitral annular disjunction, replacement fibrosis by late gadolinium enhancement, and mechanical dispersion. In this review, we briefly discuss how multimodality cardiac imaging can be applied to identify MVP patients with high risk of sudden cardiac death and complex ventricular arrhythmias.

## Introduction

Mitral valve prolapse (MVP) has received renewed attention due to several studies documenting its association with scar substrate and increased risk of complex ventricular arrhythmias (VA) and sudden cardiac death (SCD) (1–3). Other important complications related to MVP are mitral regurgitation, infective endocarditis, cerebral embolic events, and heart failure (4). Historically, a mid–late systolic click was first described to be associated with mitral regurgitation by Barlow and Pocock (5). Later, Criley et al. demonstrated that this click associated with mitral regurgitation is due to mitral valve prolapse (6).

The current echocardiographic definition of MVP is displacement of one or both mitral leaflets in the left atrium below the annulus by at least 2 mm in the parasternal long-axis view (7). The prevalence of MVP in the general population is currently thought to be around 2–3% (8, 9). However, historically, the reported prevalence of MVP has been described to be as high as 17–35% (10, 11). These discrepancies are related to initial reports which defined MVP as the presence of 2 mm leaflet displacement in any apical echocardiographic view (two-, three-, or four-chamber). This led to an overdiagnosis of MVP primarily due to the assumption that the mitral valve is a planar structure, which was later refuted by the pioneering work of Levine et al. in the latter half of the 1980s (7, 12). Most of the MVP cases are sporadic in nature; however, studies have also demonstrated a genetic association (13, 14).

This review will focus on MVP due to myxomatous valvular degeneration. In myxomatous valve degeneration, diffuse thickening, redundancy, and elongation of mitral valve leaflets occur in association with an abnormality of the chordae. Barlow's disease and fibroelastic deficiency (FED) are ends of the myxomatous MVP spectrum. Barlow's disease is often found in middle-aged patients with severe annular dilatation, excessive valvular tissue, thickened or thin chordae, and prolapse of most of the mitral leaflet segments (15). On the other hand, FED is more often found in elderly patients with less severe dilatation of the mitral annulus and thickening of valve tissue predominantly within the prolapse segments with thinner non-prolapse segments and chordae (15).

In addition to diagnosing MVP and quantifying associated mitral regurgitation, cardiac imaging is emerging as a tool to risk stratify for malignant VA and SCD. In this review, we briefly discuss recent studies which have identified cardiac imaging-based risk markers of these adverse events in patients with MVP.

## Incidence of Malignant Ventricular Arrhythmia and Sudden Cardiac Death

There are two broad sets of data on MVP and SCD in the literature: one is from autopsy studies of subjects with SCD and the other is from observational studies on the incidence of SCD among living patients with MVP. The cause of SCD in MVP patients is assumed to be malignant VA (16, 17). In fact, a recent study identified a strong association between VA burden and increasing mortality risk among patients with MVP (18). Notably, data on the true incidence of MVP in SCD patients from autopsy studies is underestimated (19). Many studies despite the confirmed presence of MVP in SCD patients categorized them as “undetermined” (19). A meta-analysis found a significant heterogeneity among autopsy studies investigating the cause of SCD (3). They reported that MVP was present in 11.7% of SCD patients without other structural heart disease (3). Basso et al. reported MVP to be the third most common finding in SCD autopsy cases of young subjects (<40 years) with a prevalence of 12% (20). Another study from France reviewed negative medicolegal autopsies of 1,000 adults (<65 years) for cardiac causes and found 125 subjects had MVP (21). All these suggest MVP to be highly prevalent among SCD cases.

Among living patients with MVP, there is wide variability in the limited data reported on the incidence of SCD in MVP. A meta-analysis reported the incidence of SCD to be 0.14% from two observational studies comprising 520 patients (3). Most likely, this is underreported due to lack of studies where data have been collected systematically. A recent large prospective study (*N* = 177), where MVP was diagnosed by cardiac magnetic resonance (CMR), reported 1.2% annual event rate of SCD or malignant VA (2). The reported incidence of premature ventricular extrasystole (PVC) or ventricular tachycardia is very high on 24-h ambulatory Holter monitoring among patients with MVP with a recent study of consecutive patients (*n* = 595) with MVP and 24-h ambulatory Holter monitoring finding nearly 43% having either PVC burden >5% or ventricular tachycardia (18).

## Risk Stratification Using Clinical Variables

Demographically, MVP patients who suffer from SCD are described to be younger and more often females (1, 17, 22). T-wave inversion/ST-segment depression and QTc prolongation are often described in living patients with MVP (1, 18, 23, 24). Furthermore, a recent large series (*n* = 595) found the presence of T-wave inversion/ST-segment depression to be associated with a nearly 2-fold increased risk of mild–moderate VA and 8-fold increased risk of severe VA (18). Basso et al. found T-wave inversion in the inferior leads of antemortem electrocardiogram in 10 out 12 patients with MVP who suffered from SCD (1).

## Application of Cardiac Imaging for Risk Assessment

**Figure 1:**
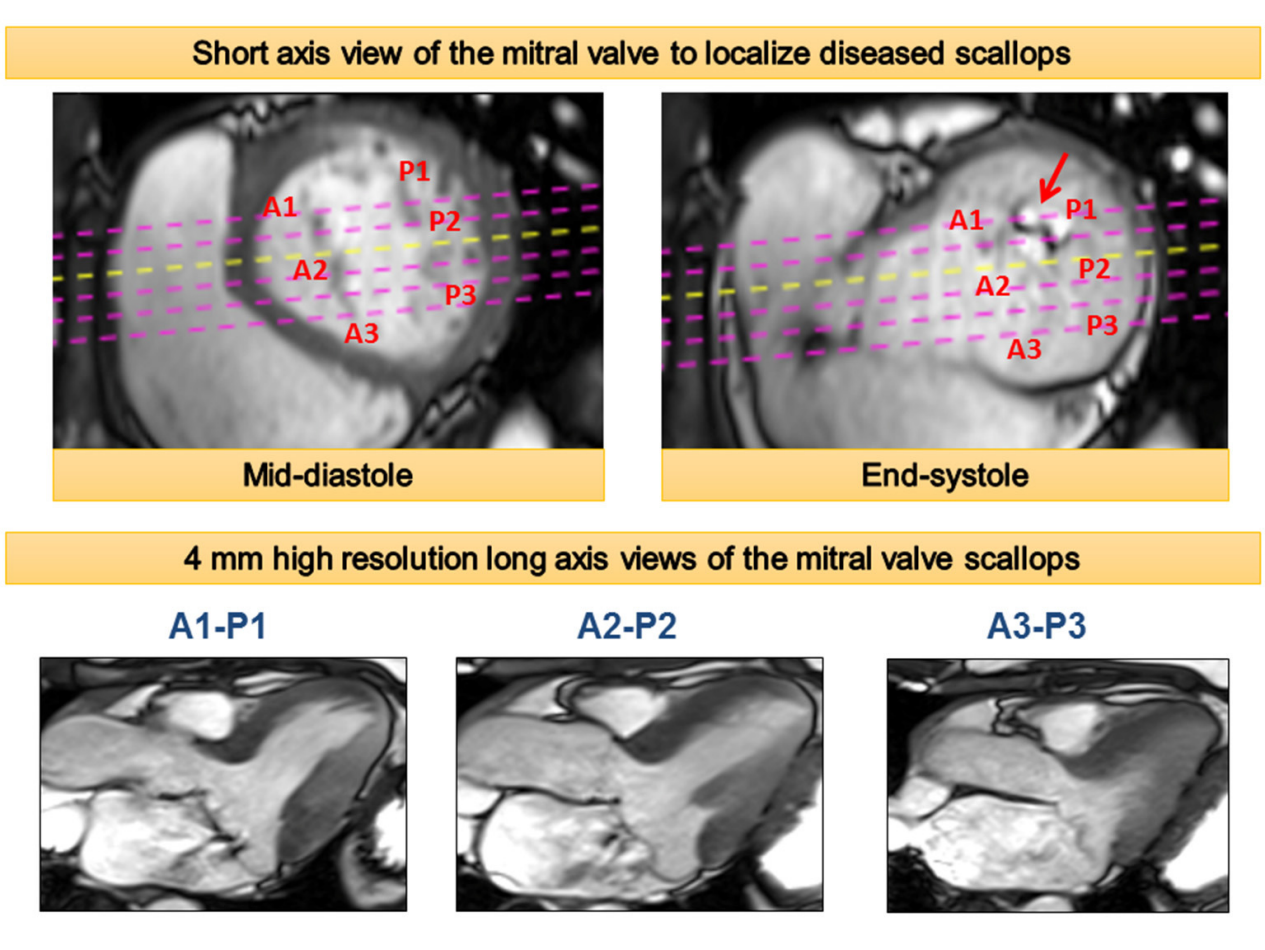
Cardiac magnetic resonance approach to assessment of mitral valve morphology and mechanism of mitral regurgitation. A stack of high-resolution three-chamber cine views perpendicular to the short axis of the valve are acquired to investigate individual mitral valve scallops as demonstrated in the artwork.

The diagnosis of MVP is fundamentally made by cardiac imaging—primarily echocardiography. In addition to establishing the diagnosis of MVP, echocardiography can be used to quantify associated mitral regurgitation and left ventricular (LV) remodeling. Recently, CMR has gained traction for the assessment of valvular heart disease. It can provide more accurate quantification of regurgitation volume and LV remodeling (25). This is in part due to its ability to compute LV volumes without the need for geometric assumptions. Taking this into notice, the recent American Society of Echocardiography recommends referral for CMR for quantification of mitral regurgitation in scenarios when there are discordances in the various echocardiographic parameters (26). For diagnosing MVP, the same criteria of 2 mm leaflet displacement in a three-chamber long-axis view can be applied to CMR (27). CMR can also identify the prolapsed scallops within each leaflet using a stack of cine images (Figure 1) (27). Further, CMR is ideally suited for risk stratification of VA in patients with MVP due to its unique ability to non-invasively identify focal myocardial fibrosis.

### Valve Morphology

The presence of bileaflet prolapse on echocardiography with redundant valve leaflets is one of the major markers of increased incidence of VA and SCD among patients with MVP. It is reported that nearly 70% of cases with MVP and SCD had bileaflet prolapse, while only 26% had posterior leaflet prolapse (17). A similarly high prevalence of bileaflet prolapse (70%) among autopsy cases of MVP was noted (1). Redundancy of mitral valve leaflets, a typical characteristic of myxomatous mitral valve prolapse, is also described in autopsy cases of SCD with MVP. A meta-analysis reported that 72 out of 73 confirmed SCD cases with MVP had redundant leaflets (17).

### Myocardial Fibrosis

**Figure 2:**
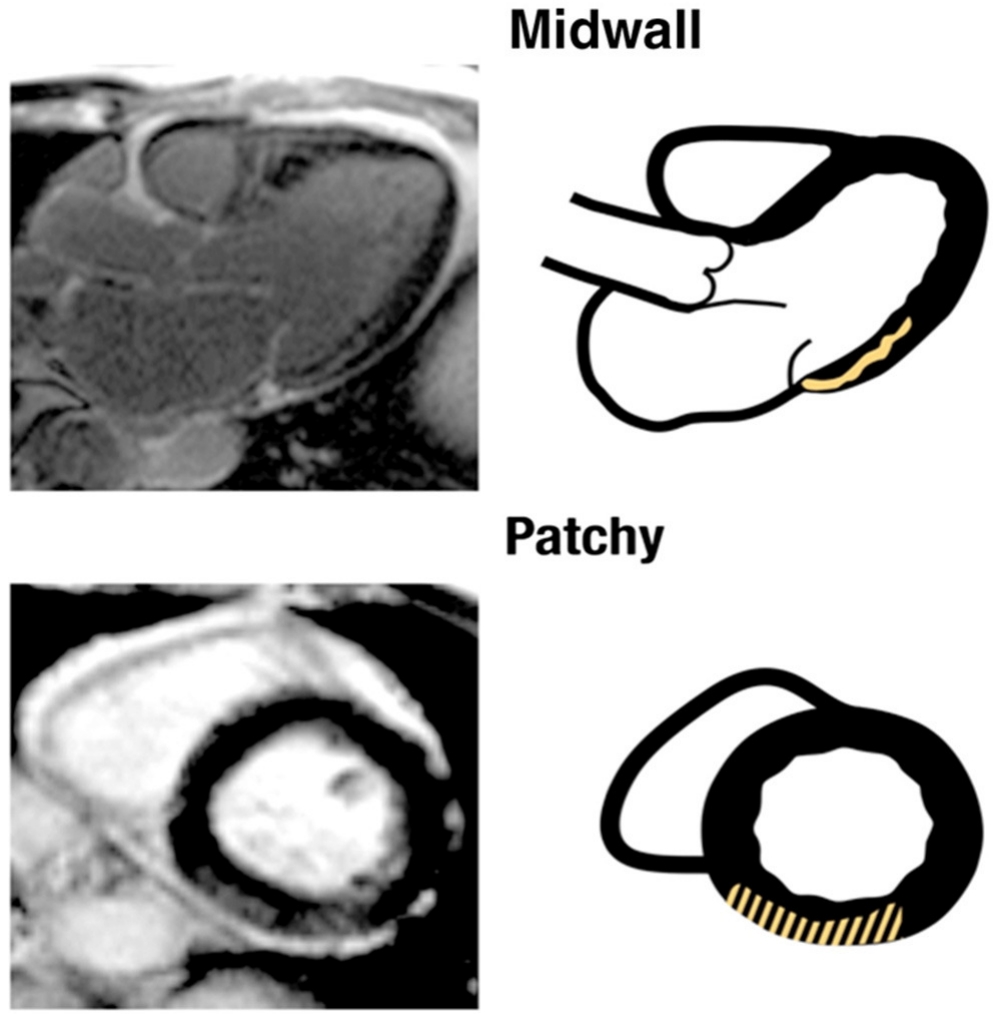
This artwork by Kitkungvan et al. (2) shows the type and regional distribution of scar among patients with mitral valve prolapse (adapted with permission from Elsevier and Copyright Clearance Center).

Autopsy studies have demonstrated the presence of myocardial fibrosis among patients with MVP (22, 28). However, due to small sample size and lack of systematic analysis, knowledge gaps remain. By contrast, CMR allows non-invasive assessment of focal replacement fibrosis in the LV by utilizing late gadolinium enhancement (LGE). Using CMR, several studies have reported the presence of LV myocardial fibrosis among patients with MVP (1, 2, 27, 29). These studies show that fibrosis often occurs in the posteromedial papillary muscle and adjacent LV wall segment (e.g., inferior and inferolateral LV walls). Besides being focal, LGE fibrosis is typically of mid-myocardial or patchy pattern in patients with MVP (Figure 2) (2). This focal distribution of fibrosis is unique to patients with MVP and cannot be ascribed to LV remodeling due to mitral regurgitation (2). One of the proposed mechanism of this regional fibrosis is repeated systolic papillary muscle traction (30, 31). A paradoxical movement of papillary muscle tip during systole is described; while the LV annulus moves toward the apex, the papillary muscle tip moves in the opposite direction toward the LA (31). It is hypothesized that excessive and repeated papillary muscle traction by the prolapsing leaflets leads to the development of LV fibrosis (32). However, it is difficult to rule out whether these patients have a distinct underlying cardiomyopathy causing regional fibrosis.

Several studies provide antecedent association to the presence of this regional myocardial fibrosis to complex VA (2, 27, 33) (Table 1). Han et al. demonstrated that 8/10 MVP patients with complex VA had myocardial fibrosis in the region of papillary muscle (27). In another study, Kitkungvan et al. prospectively followed a large series of MVP patients (*n* = 177) over a median period of 3.7 years. In his cohort, eight patients had either ventricular tachycardia (VT) or aborted SCD (2). Of these, five patients had mid-wall or patchy myocardial fibrosis (non-ischemic pattern) in the inferobasal region of the LV.

**Table 1:** Studies describing imaging risk factors in patients with MVP.

**Studies**	**Study design**	**Total patients**	**Imaging modality**	**Arrhythmias**	**Imaging findings**
**Myocardial fibrosis**					
Han et al. (27)	Retrospective	16	CMR	8 patients (NSVT or couplets)	10 had PM fibrosis. All 8 with VA had fibrosis
Basso et al. (1)	Prospective	44	CMR	30 patients (VF = 2, VT = 1, NSVT = 27)	30 patients with LGE at least one region (25 PM, 16 inferolateral mid-wall, 24 inferolateral basal wall). 28 out 30 with VA had fibrosis
Bui et al. (33)	Retrospective	41	CMR	14 patients had NSVT (out of 32 with Holter data)	11 patients had PM fibrosis; 5 out of 14 patients with VA had PM fibrosis
Kitkungvan et al. (2)	Prospective	177	CMR	8 patients had inducible VT, sustained VT, or sudden cardiac death over 3.7 years follow-up.	65 patients had myocardial fibrosis. The most common locations were basal or mid inferolateral wall and basal inferior wall. Arrhythmic event rate of 7.7% in MVP patients with replacement fibrosis vs. 2.7% in MVP patients without replacement fibrosis
**Mitral annular disjunction (MAD)**					
Eriksson et al. (43)	Not specified	32	TEE	No VA data	31 of 32 patients had MAD with mean MAD of 10 ± 3 mm
Carmo et al. (47)	Retrospective	38	TTE	NSVT by Holter (number not specified)	21 patients had MAD. Average MAD was 7.4 ± 8.7 mm. Patients with NSVT had larger mean MAD. MAD ≥8.5 mm was associated with NSVT
Lee et al. (45)	Prospective	101	TEE	No VA data	42 patients had MAD with a median MAD of 8.9 mm
Dejgaard et al. (46)	Cross-sectional	116 patients with MAD, of which 90 had MVP	115 had TTE and 83 patients had CMR	26 had NSVT and 14 had sustained VT or SCD	64 patients with MVP had MAD
Essayagh et al. (44)	Prospective	89	CMR	NSVT, number not specified	31 patients had MAD with a mean MAD of 8 ± 4 mm. Patients with MAD had more often NSVT
**Mechanical dispersion**					
Ermakov et al. (54)	Retrospective	59	TTE	32 patients had complex VA; among these, 9 had secondary prevention ICD	Patients with MVP and VA had higher mechanical dispersion in comparison to those with MVP and no VA (mean 59 ± 21 vs. 43 ± 12, *P* < 0.001)
**Pickelhaube sign**					
Muthukumar et al. (58)	Retrospective	21	TTE	10 patients had either VT or VF	12 (57%) patients had Pickelhaube sign with late high-velocity systolic spike (≥16 cm/s) with higher incidence of malignant VA (67 vs. 22%)

*CMR, cardiovascular magnetic resonance imaging; VT, ventricular tachycardia; NSVT, non-sustained ventricular tachycardia; VF, ventricular fibrillation; PM, papillary muscle; LGE, late gadolinium enhancement; VA, ventricular arrhythmia; TTE, transthoracic echocardiography, TEE, transesophageal echocardiography; MVP, mitral valve prolapse*.

It is well-documented in the literature that the presence of scar can lead to the genesis of ventricular arrhythmia. Some viable tissue embedded in dense scar can form slow conduction channels which can generate a re-entry circuit causing arrhythmias (34). However, further evidence is needed to substantiate that this regional fibrosis reported among patients with MVP is the precipitating cause of ventricular arrhythmia. These patients may have some underlying electrical abnormalities like QTc prolongation which is causing the VA. Therefore, further evidence is provided by electrophysiological studies to validate this hypothesis. ECG, the most commonly available electrophysiological tool, has demonstrated that VA are of right bundle branch block morphology among patients with MVP indicating that arrhythmias originate in the LV (1). Moreover, in a study where ablation was attempted among MVP patients who survived cardiac arrest or have symptomatic ventricular extrasystoles, the papillary muscle or subvalvular mitral apparatus region was the primary focus for ventricular fibrillation or dominant ventricular extrasystole (35). An earlier study using vectorcardiography of surface electrocardiogram reported that the origin of ventricular extrasystole in patients with MVP is in the posterobasal region of the LV (36). These concordances of the trigger site for arrhythmias and the presence of myocardial fibrosis in the same region are unlikely to be mere coincidence. One hypothesis for the genesis of arrhythmias in these patients is that morphological changes around the mitral annular plane and papillary muscle, combined with excessive motion due to mitral prolapse in this region, cause electrical instability leading to VA (37, 38).

More corroborating evidence is provided by autopsy studies. Basso et al. systematically investigated all young adults (<40 years) with SCD (*n* = 650) in the Veneto region in Italy between 1982 and 2013 (1). They identified 43 patients with MVP. All 43 of these patients had patchy fibrosis in the region of the posterior papillary muscle and the LV wall adjacent to it. They further investigated 30 living patients with MVP with evidence of complex VA and found 28 (93%) of these patients had myocardial fibrosis in the region of papillary muscle with or without involvement of the inferobasal region of the LV wall.

Considering these findings, the prior theory of an occult MVP cardiomyopathy has evolved into that of a localized mechanical injury of the myocardium such that MVP patients have unique focal fibrosis which is the substrate of SCD and complex VA (38). Further evidence against an occult cardiomyopathy was provided by a recent investigation from our group which noted that accounting for the volume load from the prolapse volume (e.g., the volume of blood above the left ventricular muscle but below the prolapsing mitral leaflets in the ventricularized portion of the left atrium) reconciles the disproportionate LV enlargement that is noted in some MVP patients (39). However, further study is needed, and this is still an area of active investigation.

Cardiac imaging, specifically CMR, may help in risk stratification by assessing fibrosis. In fact, a study demonstrated diffuse LV fibrosis determined by T1 mapping using CMR is also associated with increased risk of complex VA among patients with MVP (33). In this study, the authors found a significant shorter post-contrast T1 time among patients with MVP and complex VA in comparison with those without VA. However, further studies are required to understand if this reduced T1 time is a preceding marker of future LV fibrosis or represents some underlying cardiomyopathy which makes the patients susceptible to VA.

### Mitral Annular Disjunction

**Figure 3:**
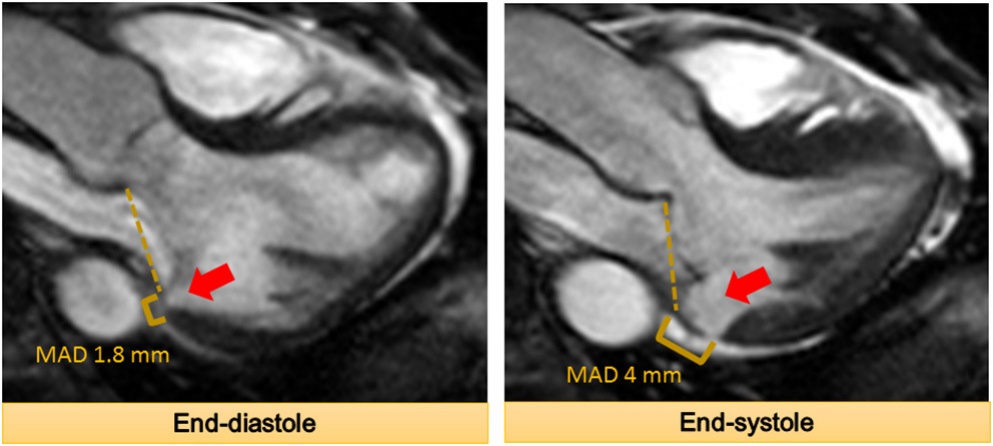
The mitral annular disjunction (MAD) at the inferolateral wall denoted by the arrow is demonstrated. It should be noted that MAD increases from end-diastole to end-systole.

Mitral annular disjunction (MAD) is a separation of the posterior mitral annular hinge point from the LV wall while the attachment to the atrium and mitral valve is preserved (Figure 3). The detachment or disjunction allows the excessive motion of the mitral valve during systole. It was first described by Bharati et al. in 1981 in a case report. They reported that the annulus was elongated in patients with MVP and the valve was anchored to the atrial side creating a separation between the atrium and LV (40). Subsequently, Hutchinson et al. performed an autopsy study of the hearts of patients with SCD (*n* = 900) (41). They described that 23 (92%) out of 25 hearts with MVP and SCD had MAD, whereas only 6% of the hearts with other causes of SCD had this displacement. However, a contradictory report challenging this concept of MAD in patients with MVP was published soon after (42). Angelini et al. studied 13 hearts obtained from necropsy (seven normal and six with MVP). They concluded that there is variation in the atrioventricular junction in all the hearts. They proposed that the appearance of separation is in fact produced by thickening of the valve leaflet at the hinge point and not actual displacement (42).

Recent studies have reported MAD among patients with MVP using echocardiography (two-dimensional and three-dimensional) and CMR (43–47) (Table 1). A systematic review of these studies showed that nearly 40–60% of patients with MVP have MAD (48). Studies have described a mean MAD varying from 5 to 10 mm in the long-axis view (parasternal or apical) using different imaging modalities (43–45, 47). In fact, a recent study postulated MAD as an independent entity which results in increased risk of VA (46). In this study, of 116 patients, 115 had MAD by echocardiography and 1 by lone CMR. The authors reported that nearly 1 out of 4 patients with MAD did not have concomitant MVP, suggesting that MAD is not necessarily exclusive to patients with MVP. MAD can be observed at different locations on the mitral annulus. However, they observed that MAD is associated with increased risk of VA when it is present at the posterior wall (46). More recently, a new approach to measure MAD using cardiac computed tomography (CCT) is described. In this study, the authors retrospectively studied patients with MVP (*n* = 90) for the presence of MAD (49). They noted MAD detectable by CCT in ~20% of the patients with severe mitral regurgitation scheduled for mitral valve surgery. This proof-of-concept study highlights a potential role of CCT due to its high spatial resolution, albeit modest temporal resolution.

Further study and understanding of the concept of MAD are warranted. There is a need to further characterize changes in MAD between systole and diastole. Mechanistic data is required as to why MAD may cause an increased risk of VT. Moreover, the impact of surgical mitral valve repair on MAD is unknown and warrants further investigation.

### Mechanical Dispersion and Longitudinal Strain

Mechanical dyssynchrony is associated with VA in patients with cardiomyopathies (50, 51). In an experimental model, it is elucidated that mechanical dyssynchrony is associated with the expression of certain proteins which makes the heart susceptible to complex VA (52). Mechanical dyssynchrony can be quantified with multiple techniques, including deformation imaging and tissue Doppler imaging (53). Mechanical dispersion is one of the methods to quantify mechanical dyssynchrony. It is derived using the standard deviation of time-to-peak of shortening of 18 LV segments using deformation imaging in the 3 apical views. A recent study investigated the presence of mechanical dyssynchrony among patients with MVP and found that patients with MVP and VA had significantly higher mechanical dispersion compared with those with MVP and no VA (54). This increased dispersion can be related to myocardial fibrosis observed in MVP patients. Regional fibrosis causes delayed contraction in segments with fibrosis leading to increased mechanical dispersion. This could be another less costly but effective approach to identify MVP patients at high risk of SCD and VA using echocardiography.

Besides mechanical dispersion, the magnitude of longitudinal strain can be used to identify early signs of subclinical cardiomyopathy among patients with MVP. One study described decreased longitudinal LV strain in patients with classical MVP (>5 mm leaflet thickness) (55). Another study demonstrated that basal LV segments have higher longitudinal strain in patients with Barlow's disease indicating hypermobility, but overall, this study did not find any difference in global LV function between patients with Barlow's disease and FED (56). More recently, a study showed that regional post-systolic shortening is observed more often among MVP patients with malignant VA (57). However, this study comprises only 44 patients with just five of them having malignant VA. Therefore, the findings need to be corroborated in larger settings.

### Pickelhaube Sign

**Figure 4:**
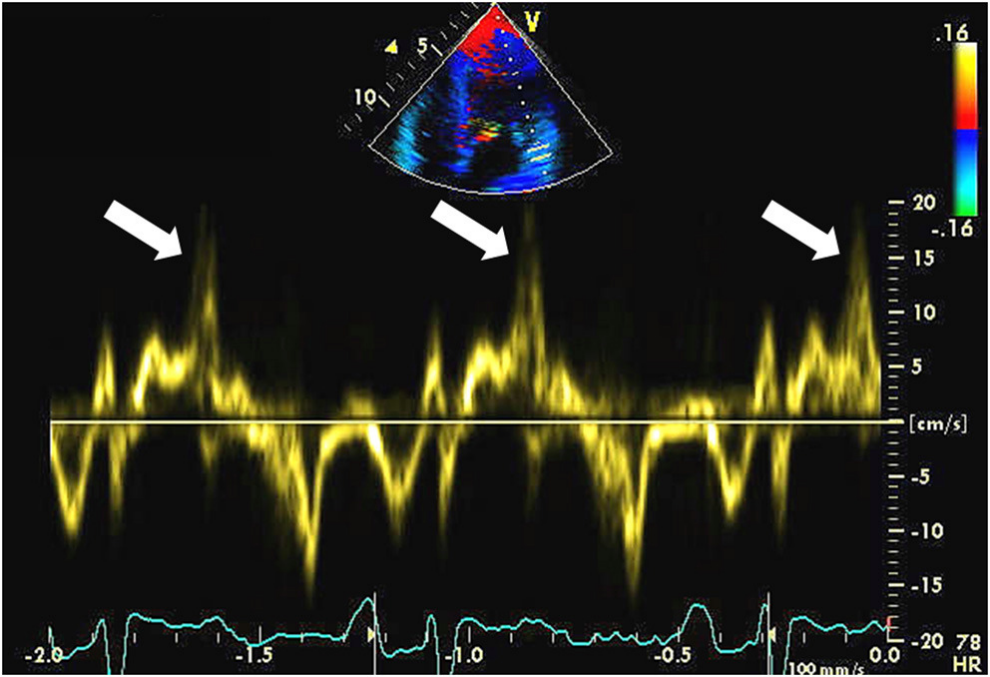
An echocardiographic example of the “Pickelhaube sign” which reflects increased velocity at the lateral mitral annulus in mid-systole to late-systole. The velocity spike is denoted by a white arrow.

Muthukumar et al. using pulsed-wave Doppler measured mitral annular velocity in a series of 21 patients with MVP (58). They noted a distinctive spike mid-systole to late-systole at the lateral mitral annulus (>16 cm/s) in 12 of 21 patients (Figure 4) (58). Of these 12 patients, eight patients suffered from malignant VA at follow-up. No similar spike of increased velocity was observed at the medial mitral annulus. The authors hypothesized that the tugging of the posteromedial papillary muscle of the prolapsing myxomatous mitral leaflet causes the basal LV wall to pull toward the apex with increased velocity. Pickelhaube is the term used to describe the German military helmet which has a spike on top; therefore, this was used to describe the distinctive spike. Later, the same group showed that the Pickelhaube sign in patients with MVP is found with higher frequency at the posterolateral annulus in the apical long-axis view (59). This is a novel and relatively simple approach. Larger studies are required to validate the findings.

## Risk Stratification

**Figure 5:**
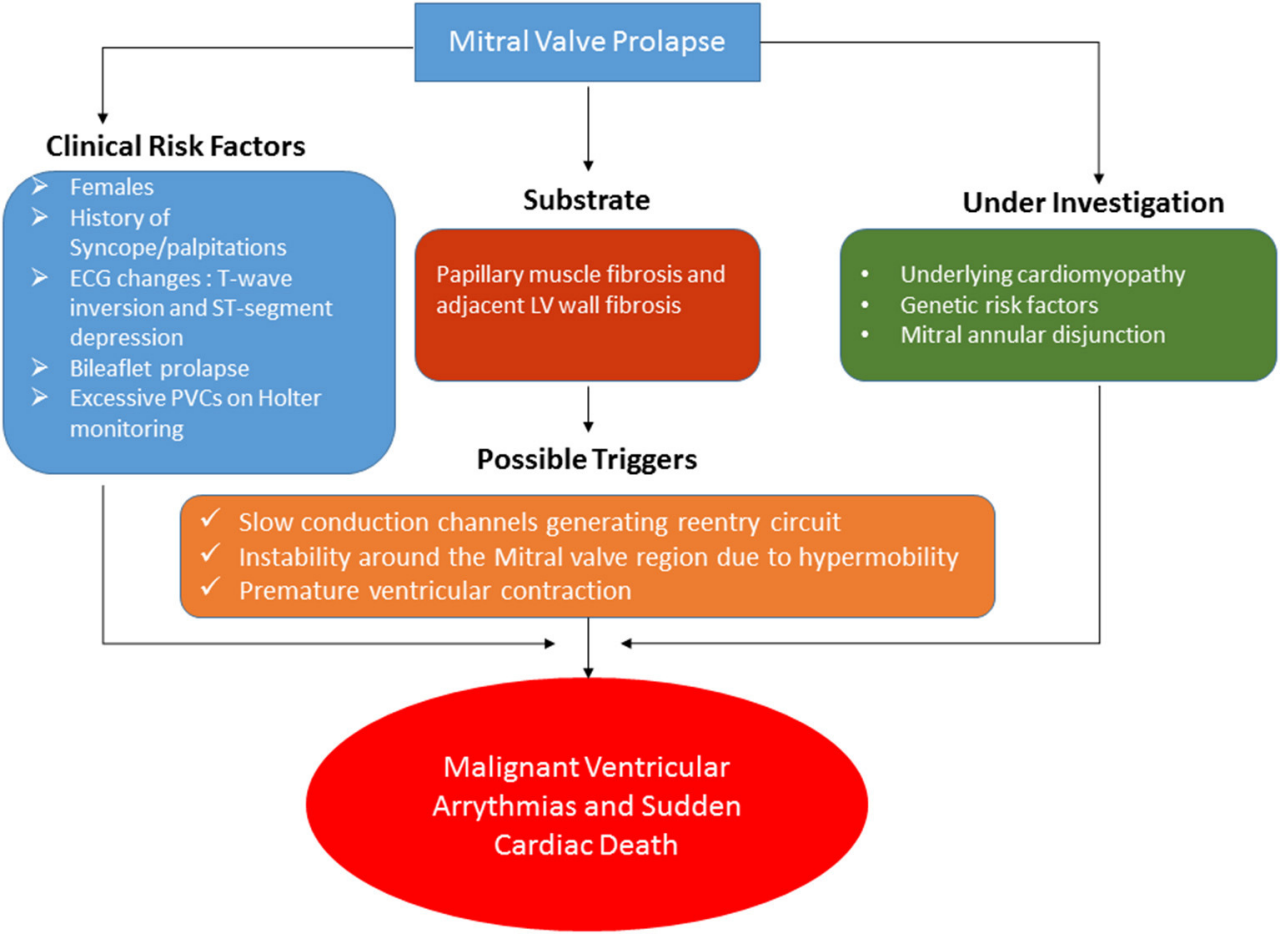
The clinical risk factors, mechanism, and hypothetical factors still under investigation related to the potential increased risk of malignant ventricular arrhythmias and sudden cardiac death among patients with MVP are illustrated.

MVP is widely prevalent in the community. Although the risk of SCD may be low, it is of serious concern due to its devastating consequences. A risk stratification strategy is needed to identify patients at high risk of this consequential complication. Figure 5 summarizes the factors associated with SCD along with the likely mechanism. Among the clinical variables which suggest increased risk are as follows: female sex, bileaflet prolapse, and ECG changes (ST and T-wave changes in the inferior leads) (17, 38). Besides these, other risk factors can be clinical history of palpitations or unexplained syncope. A 24-h Holter monitoring may be considered to estimate the burden of VA. If any of this is present, a CMR can be performed for the assessment of LV fibrosis, especially in the posterior subpapillary region. As explained before, most likely, this LV fibrosis serves as the substrate for the VA. Slow conduction channels embedded in this fibrotic region along with hypermobility of the basal segments and a PVC are possible triggers leading to a full-fledged malignant VA. The presence of a concomitant underlying cardiomyopathy and MAD are other hypothetical factors which may lead to increased risk of SCD and VA.

## Future Directions

There are many small series demonstrating SCD and malignant VA among patients with MVP (1, 2). However, there is a need for a large multicenter study where patients are enrolled prospectively and systematically followed at regular intervals with Holter monitoring and periodic follow-up with multimodality imaging—particularly echocardiography and CMR. This will provide insights into the true risk of SCD and malignant VA among patients with MVP. Imaging may aid by providing high-risk markers which may identify patients who warrant closer follow-up. Moreover, imaging findings along with clinical and genetics/molecular research can be combined to develop a scoring system which can eventually be applied to identify patients at high risk of SCD and considered for implantable cardioverter defibrillator device as primary prevention. The most recent ACC/AHA/HRS guidelines from 2017 note the increased risk of VA among patients with MVP and a potential association with SCD, but there are no specific recommendations made for therapeutic intervention (60). Among the imaging risk markers, factors which should be investigated are the role of LV fibrosis in the posterior subpapillary region, MAD, T1 mapping, longitudinal strain, and Pickelhaube signs. The role of other imaging modalities, such as positron emission tomography (PET) should also be considered. A recent study using PET demonstrated an increase uptake of FDG with concurrent LGE by CMR in patients with MVP, raising the possibility of an inflammatory or ischemic component in these segments among asymptomatic patients with MVP (61). Further studies will provide more insight into the role of PET in risk stratification of MVP patients.

## Conclusions

MVP is a widely prevalent disease and studies have shown the increased risk of SCD and complex VA in these patients. Several imaging markers are shown to be related to this arrhythmic risk. Aside from its role as a cornerstone for identifying MVP, echocardiography can serve as a gatekeeper for further investigations if bileaflet prolapse or signs of any cardiomyopathy are noted. The strength of CMR is its ability to perform tissue characterization to identify focal myocardial fibrosis in the posterior papillary muscle and adjacent LV wall. The presence of fibrosis has one of the most compelling mechanistic basis and supporting evidence in the literature. Patients presenting with syncope, pre-syncope, palpitations, or evidence of VA should be investigated with CMR for further risk stratification.

## Author Contributions

All authors listed have made a substantial, direct and intellectual contribution to the work, and approved it for publication.

## Conflict of Interest

The authors declare that the research was conducted in the absence of any commercial or financial relationships that could be construed as a potential conflict of interest.
